# Non-Targeted Metabolomics Analysis Reveals the Inhibition Mechanism of Ozone Treatment on Postharvest Blue Mold in *Angelica sinensis*

**DOI:** 10.3390/foods15030493

**Published:** 2026-02-01

**Authors:** Jihui Xi, Kunhao Jiang, Huali Xue, Yang Bi

**Affiliations:** 1 College of Food Science and Engineering, Gansu Agricultural University, Lanzhou 730070, Chinabiyang@gsau.edu.cn (Y.B.); 2 College of Science, Gansu Agricultural University, Lanzhou 730070, China

**Keywords:** *Angelica sinensis*, ozone, *Penicillium polonicum*, blue mold, metabolomics

## Abstract

*Angelica sinensis* is susceptible to blue mold caused by *Penicillium polonicum* during storage. The metabolic mechanisms of O_3_ as a fungicide in controlling blue mold caused by *P. polonicum* in *A. sinensis* remain unclear. This study investigated the effects of O_3_ treatment on the physiology, pathology, and functional active ingredients of *A. sinensis* and analyzed its impact on metabolites and metabolic pathways associated with *P. polonicum* infection. The results indicated that O_3_ inhibited the occurrence of blue mold, maintained the content of ferulic acid and ligustilide, and suppressed the quality deterioration of *A. sinensis*. Metabolomics analysis revealed that O_3_ enhances antioxidant capacity by up-regulating the tricarboxylic acid (TCA) cycle and increasing resistance to fungal invasion by up-regulating the phenylpropanoid biosynthesis pathway. Collectively, O_3_ treatment improves the quality of postharvest *A. sinensis*, which provides a theoretical foundation for the application of O_3_ in fresh postharvest storage for *A. sinensis*.

## 1. Introduction

*Angelica sinensis* is commonly known as dang gui, dong quai or toki [1] and is a plant resource for both medicine and foodstuff that has a variety of health benefits, such as tonifying blood, activating blood circulation, regulating menstruation, relieving pain, and moistening the intestines [2]. The Gansu Province of China is the dominant production area of *A. sinensis* [3]*,* with the annual output accounting for 70% to 90% of the total national output [4]. Modern medical research has demonstrated that fresh Chinese herbs have a higher medicinal composition and medicinal value than dried decoction pieces [5]. For instance, Xiong et al. found that the volatile oil content of *Pogostemon Cablin* in dried product was significantly lower than that in its corresponding fresh product [6]. Zhu et al. showed that the amino acid content of the processed product of *Radix Psammosilenes* was significantly reduced compared with that of its fresh product [7]. The polysaccharides in fresh *A. sinensis* can inhibit the release of reactive oxygen species, thus protecting macrophages [8]. The content of ligustilide in fresh *A. sinensis* is twice as much as that of the dried decoction pieces [9]. Ligustilide has antioxidant, anti-neuroinflammation, and anti-cerebral infarction clinical effects [10]. Ferulic acid has pharmacological activities such as inhibition of platelet aggregation, anti-thrombosis and cardioprotection [11]. Furthermore, senkyunolide H and senkyunolide I can up-regulate the expression of related tight junction proteins, thereby protecting cells from OGD/R-induced injury [12], while senkyunolide A and coniferyl ferulate are recognized for their anti-inflammatory activities [13]. Notably, ferulic acid and ligustilide are specified as index components for quality control of *A. sinensis* in the 2020 edition of the *Chinese Pharmacopoeia* [14].

With the development of the medicinal value of *A. sinensis* and its inclusion in the list of medicinal and food homology ingredients [15], the market for fresh *A. sinensis* demand is rapidly increasing, resulting in a growing area for *A. sinensis*. However, *A. sinensis* is harvested in late autumn with low temperatures and high humidity [16]. Blue mold caused by *Penicillium polonicum* is the prevalent disease during the storage stage after harvest, which not only seriously impacts the quality of *A. sinensis* and reduces the market value but, in severe cases, can also accumulate mycotoxin, posing a health threat [17].

At present, the storage of fresh *A. sinensis* mainly depends on traditional sulfur fumigation and shade drying, although sulfur fumigation can prevent the occurrence of blue mold [18]. However, sulfur fumigation can alter the active ingredient of Chinese herbs, leading to changes in the pharmacodynamic components, and even cause toxic reactions. Sulfur fumigation can also lead to harmful residues in Chinese herbs, and if sulfur dioxide exceeds the standard, it is harmful to human health [19]. Chemical fungicides are also adopted to manage the postharvest disease of *A. sinensis*; however, the long-term application of chemical fungicides can lead to the emergence of drug-resistant strains of pathogens, pollute the environment, and jeopardize human health [20]. Therefore, it is particularly important to develop a new safe and green strategy for the prevention and control of the postharvest disease of *A. sinensis*.

O_3_, as a strong oxidant, is widely applied for food storage and preservation [21]. Han et al. found that O_3_ treatment could effectively maintain the hardness, color and soluble solid content of *Black Mulberry* and reduce its decay rate, respiratory strength and polyphenol oxidase activity [22]; da Silva et al. suggested that O_3_ treatment could control the occurrence of postharvest anthracnose in *Papaya* and extend its shelf-life [23]; De Santi et al. found that O_3_ treatment inhibited postharvest decay of *Garlic* without affecting the organoleptic quality of *Garlic* [24]. These studies indicate that O_3_ is more environmentally friendly and safer than sulfur fumigation and chemical fungicides. Therefore, O_3_ could be a promising candidate for controlling blue mold of *A. sinensis* during postharvest storage.

Non-targeted metabolomics analysis is one of the most commonly adopted technologies for metabolomics studies and is crucial for a comprehensive understanding of cellular metabolism and for the identification of metabolites/pathways [25]. Non-targeted metabolomics is also widely employed for the detection of differential metabolites [26]. Liquid chromatography coupled with high-resolution mass spectrometry (LC-MS) allows for the detection of metabolites with high quality and precision for a wide range of chemical properties [27]. Since *A. sinensis* is a complex organism consisting of thousands of metabolites [2], a liquid chromatography–mass spectrometry (LC-MS/MS)-based metabolomics approach can be used to analyze the effects of O_3_ treatment on metabolites and the metabolic pathways of disease resistance in *A. sinensis* infected with *P. polonicum*.

In this study, we investigated (1) the effects of O_3_ treatment on the postharvest respiratory intensity, color change, weight loss, and functional active ingredients of *A. sinensis*, as well as disease control, and (2) the metabolic responses of O_3_ treatment on the disease resistance of *A. sinensis* infected by *P. polonicum*. Specifically, *A. sinensis* was inoculated with *P. polonicum*, was fumigated using O_3_, and then stored for different periods; physiological and pathological changes were observed and recorded, and tissues from the same part of *A. sinensis* under different treatment conditions were collected for metabolomic analysis using a liquid chromatography–mass spectrometry (LC-MS/MS)-based method. The effects of O_3_ treatment on metabolites and pathways related to disease resistance in *A. sinensis* were analyzed. This study will help to reveal the effects of O_3_ treatment on metabolites and disease resistance during *P. polonicum* infection of *A. sinensis* and provide a theoretical foundation for postharvest blue mold control in *A. sinensis*.

## 2. Materials and Methods

### 2.1. Sample Preparation

O_3_ was prepared by an ozone generator (Aoshan Environmental Protection Technology Co., Ltd., Dalian, China), and the O_3_ content was adjusted to 2.0 mg/L. *P. polonicum* strains were isolated and purified from *A. sinensis* blue mold tissues in our laboratory and were identified morphologically and molecularly [17]. *P. polonicum* was cultured on PDA at 25 °C for 14 days, and spore suspensions were prepared at a concentration of 1 × 10^6^ CFU/mL.

*A. sinensis* was harvested from the base of an *A. sinensis* plantation in Min County, Gansu Province, China. Healthy and fresh *A. sinensis* samples without obvious mechanical damage or insect pests were selected; transported to the Laboratory of Chemistry and Biology, College of Science of Gansu Agricultural University, on the same day; washed several times until there was no soil residue, followed by sterilization with 0.1% sodium hypochlorite for 15 min; washed with sterile distilled water to remove sodium hypochlorite residue on the surface; and dried under natural conditions.

A 1 × 10^6^ CFU/mL *P. polonicum* spore suspension was kept in a 10 mL sterile spray bottle and evenly sprayed on the surface of *A. sinensis*, with a spray volume of 3 mL. The control group was sprayed with an equal amount of sterile water. The treated *A. sinensis* was placed in a sterile freshness bag (30 cm × 43 cm). The experiment comprised four treatment groups. Samples in all groups were placed in sterile freshness bags (30 cm × 43 cm), with the control treatment consisting of spraying with an equal amount of sterile water. The groups were defined as follows: the uninoculated fungal control (UF-CK) group, which was not inoculated with *P. polonicum*; the uninoculated with ozone treatment (UF-O_3_) group, which received a daily 30-minute O_3_ treatment for 7 consecutive days; the *P. polonicum*-inoculated control (PP-CK) group; and the *P. polonicum*-inoculated with O_3_ treatment (PP-O_3_) group, which also received the 7-day O_3_ treatment. After these respective pre-treatments, all groups were stored in the dark at room temperature under controlled conditions of 25 °C and 50% relative humidity (RH). The above treated samples were stored for 0, 7, 14, 21, 28, 42, and 56 days in the dark (25 °C, 50% RH).

### 2.2. Experimental Observations, Sample Design, and Metabolomics Analysis

Changes in physiological indices (respiratory intensity, chrominance, weight loss) and pathological indices (disease incidence, disease index) were observed during storage, and functional active ingredients were determined by UHPLC. The experiment employed a complete randomized design with three independent biological replicates per treatment (*n* = 3). Within each biological replicate for a given treatment, nine parallel specimens were harvested at each of the seven storage time points for the aforementioned measurements. Consequently, the entire study involved a total of 756 individual specimens (4 treatments × 3 biological replicates × 7 time points × 9 specimens per time point). For statistical analysis, data from the nine specimens within each biological replicate at a given time point were averaged to represent that replicate.

**Figure 1:**
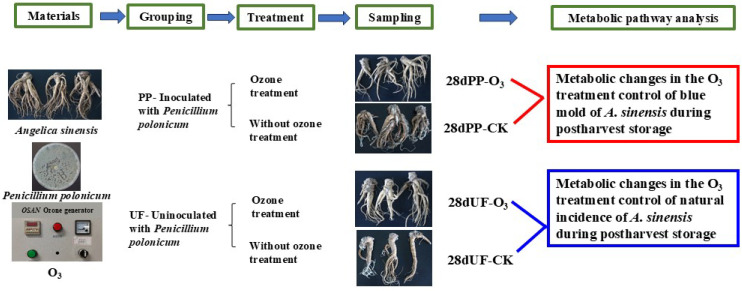
Flowchart of sample grouping, treatment, and collection. The experimental design combined two factors: inoculation status [PP (inoculated with *P. polonicum*) or UF (uninoculated)] and treatment [O_3_ (Ozone treatment) or CK (control, no ozone treatment)]. Samples from the resulting four groups were collected at multiple time points for analysis.

Metabolomics analysis of *A. sinensis* samples was conducted on the 28th day of storage; in order to balance the position effect and reduce experimental error, the sampling positions of *A. sinensis* roots in the treatment group and the control group corresponded to the same part in three replications. UF-CK indicates the control group not inoculated with *P. polonicum*, UF-O_3_ indicates the O_3_-treated group not inoculated with *P. polonicum*, PP-CK indicates the control group inoculated with *P. polonicum*, PP-O_3_ indicates the O_3_-treated group inoculated with *P. polonicum*; 28 d indicates 28 days of storage; ‘-1’, ‘-2’ and ‘-3’ indicate replication 1, replication 2 and replication 3. The samples of *A. sinensis* were taken from the control group and the treated group at day 28 of storage. The samples of *A. sinensis* were frozen in liquid nitrogen for 15 min after grinding and then quickly transferred to an ultra-low temperature refrigerator (−80 °C) for storage until further use. The experimental design and flow are shown in Figure 1. Sequencing of the *A. sinensis* metabolome was entrusted to Shenzhen Wekemo Bioincloud Co., Ltd. (Shenzhen, China).

### 2.3. Methods

#### 2.3.1. Respiratory Intensity

Following a slightly modified version of the method by Chen et al. [28], fresh *A. sinensis* tissue was placed in a 5300 mL sealed jar and left to stand for 20 min. The CO_2_ concentration was measured using a portable detector (Model RC5002, Guangzhou Rongce Electronic Co., Ltd. (Guangzhou, China), and respiratory intensity was calculated with reference to Formula (1):

Respiratory intensity/(mg/kg/h) = {[(M/22.4)] × N × [273/(273 + T)]} × [V/(m × h)](1)

In the formula, m is the mass of fresh *A. sinensis* (g); M is the relative molecular mass of CO_2_ gas; N is the CO_2_ concentration (mg/m^3^); T is the ambient temperature (25 °C); V is the volume of the desiccator (L); and h is the time of measurement (h).

#### 2.3.2. Chrominance

The chrominance of *A. sinensis* was measured in 7-day intervals using a colorimeter, and the total chrominance of *A. sinensis* was expressed as the value (E) [29]. The formula was calculated as follows:

E = (L*^2^ + a*^2^ + b*^2^)^1/2^ (2)

In this context, L*, a*, and b* represent values that are measured directly by the colorimeter.

#### 2.3.3. Weight Loss

The mass of *A. sinensis* was measured and recorded in 7-day intervals using an analytical balance, and the measurements were repeated three times for each group of samples. The weight loss of *A. sinensis* was calculated as shown in Formula (3):

Weight loss (%) = (initial mass-mass after N days)/(Initial mass) × 100%(3)

#### 2.3.4. Disease Index and Disease Incidence

Disease index and disease incidence were measured and calculated according to Formulas (4) and (5), as follows [30,31]:

Disease Index = {[sum (class frequency × score of rating class)]/[(Total number of plants × maximal disease index)]} × 100(4)

Disease incidence = (Number of the infected plants)/(the number of plants sampled) × 100(5)

For the two formulae, disease level was classified into five categories, 0, 1, 2, 3, and 4 (Table 1), according to Cao et al. [32], with minor modifications.

#### 2.3.5. Analysis of Functional Active Ingredients of *A. sinensis*

The functional active ingredients of *A. sinensis* were determined in accordance with the method of Luo [33]. The fresh *A. sinensis* was treated according to Section 2.1: 5.0 g of the same parts of the treated *A. sinensis* was sampled and placed in a mortar pre-cooled by liquid nitrogen and then quickly ground to powder under the protection of liquid nitrogen. Subsequently, 0.5 g of the powder was transferred into a centrifuge tube, and 5 mL of methanol was added to the above centrifuge tube. The mixture was then extracted by ultrasonication (220 W, 80 Hz, 25 °C) for 40 min, followed by centrifugation for 10 min at 5000*× g* at 4 °C; the resultant solution was subjected to filtration through a 0.22 μm organic membrane and subsequently analyzed using UHPLC. The content of each functional active ingredient was calculated according to the regression equation between the peak areas obtained from the UHPLC analysis (Table 2).

The chromatographic analysis was performed using a Waters quaternary ultra-high-performance liquid chromatography (UHPLC) system (Waters Corporation, Milford, MA, USA) equipped with a UV–Vis detector. The conditions were as follows: the separation column was a Waters C18 reversed-phase column (5 μm, 4.6 × 250 mm; part number WAT054275); mobile phase: 1% formic acid in water (A)–acetonitrile (B); gradient elution: 0–10 min, 20–35% B; 10–20 min, 35–60% B; 20–30 min, 60–90% B; detection wavelength: 280 nm; column temperature: 30 °C; flow rate: 1.0 mL/min; injection volume: 10 μL.

#### 2.3.6. Metabolomics Analysis

##### Metabolite Extraction

The tissue samples (100 mg) were subjected to grinding in liquid nitrogen and then transferred to an EP tube. Thereafter, 500 μL of an 80% methanol aqueous solution was added. Then, the samples were subjected to a series of processing steps, including vortex shock, incubation in an ice bath for 5 min, centrifugation (15,000*× g*, 4 °C) for 20 min, the collection of the upper layer, and the addition of mass spectrometry water to achieve a methanol content of 53%. Subsequently, the samples were subjected to a further 15,000× *g* centrifugation step at 4 °C for 20 min, after which the upper layer was collected and analyzed using an LC-MS [34]. Equal volume samples were taken from each experimental sample and mixed as quality control (QC) samples.

##### Instrument Parameters

The chromatographic conditions are as follows: the column used is a HypersilGold column (C18) (100 × 2.1 mm, 1.9 μm). The column temperature is set at 40 °C. The flow rate is 0.2 mL/min. The mobile phase consists of 0.1% formic acid, and mobile phase B is methanol. The chromatographic gradient elution procedure is delineated in Table 3.

##### Mass Spectrometry Conditions

Mass spectrometric analysis was performed on a Q Exactive™ HF (or Q Exactive™ HF-X) high-resolution mass spectrometer (Thermo Fisher Scientific, German branch, Germany) equipped with an electrospray ionization (ESI) source. The parameters of the ESI source are configured as follows: the spray voltage is set at 3.5 kV, the sheath gas flow rate is adjusted to 35 psi, the auxiliary gas flow rate is maintained at 10 L/min, the temperature of the ion transfer tube is kept at 320 °C, the ion import RF level (corresponding to the S-lens RF level) is set to 60, and the heater temperature for the auxiliary gas is established at 350 °C. The polarity is configured to operate in both positive and negative modes. The MS/MS second-level scans are conducted in a data-dependent manner.

##### Data Pre-Processing and Metabolite Identification

The raw data files generated by MS were processed using Compound Discoverer 3.3 (CD3.3, ThermoFisher) to perform peak alignment, peak picking, and quantitation for each metabolite. The main parameters were set as follows: peak area was corrected with the first QC; actual mass tolerance, 5 ppm; signal intensity tolerance, 30%; and minimum intensity. After that, peak intensities were normalized to the total spectral intensity. The normalized data was used to predict the molecular formula based on additive ions, molecular ion peaks and fragment ions. Peaks were matched with the mzCloud (https://www.mzcloud.org/ (accessed on 30 January 2024)), mzVault, and MassList database to obtain accurate qualitative and relative quantitative results. Statistical analyses were performed using the statistical software R (R version R-3.4.3), Python (Python 2.7.6 version) and CentOS (CentOS release 6.6). When data were not normally distributed, we standardized according to the formula sample raw quantitation value/(the sum of sample metabolite quantitation value/the sum of QC1 sample metabolite quantitation value) to obtain relative peak areas, and compounds whose CVs of relative peak areas in QC samples were greater than 30% were removed (106 compounds were excluded in this step), and finally, the metabolites’ identification and relative quantification results were obtained.

### 2.4. Statistical Analysis of Data and Pathway Annotation

All experiments in this study were conducted with three independent biological replicates, and the resulting data were presented as the mean ± standard error. Graphical presentations were created using Origin 2021 (OriginLab, Northampton, MA, USA). For univariate analysis, significant differences between experimental groups were assessed using independent two-sample *t*-tests in the SPSS software (version 22.0; IBM Corp., Armonk, NY, USA), with a *p*-value < 0.05 set as the threshold for statistical significance. For multivariate analysis of the metabolomics data, unsupervised principal component analysis (PCA), supervised partial least squares discriminant analysis (PLS-DA), and Orthogonal Projections to Latent Structures Discriminant Analysis (OPLS-DA) were performed using the ropls package within the R statistical environment (version 3.4.3). The validity of all supervised multivariate models (PLS-DA and OPLS-DA) was rigorously evaluated through 200 permutation tests to guard against overfitting. Following statistical analysis, the putatively identified metabolites were annotated by querying their spectral features against the Human Metabolome Database (HMDB, https://hmdb.ca/metabolites (accessed on 1 March 2024)) and LIPID MAPS (http://www.lipidmaps.org/ (accessed on 1 March 2024)). Subsequently, pathway annotation was conducted using the Kyoto Encyclopedia of Genes and Genomes (KEGG) database (https://www.genome.jp/kegg/pathway.html (accessed on 1 March 2024)) to infer biological implications.

## 3. Results

### 3.1. Effects of O_3_ Treatment on Physiological, Pathological and Functional Active Ingredients of A. sinensis

#### 3.1.1. O_3_ Treatment Decreased Physiological Metabolism

**Figure 2:**
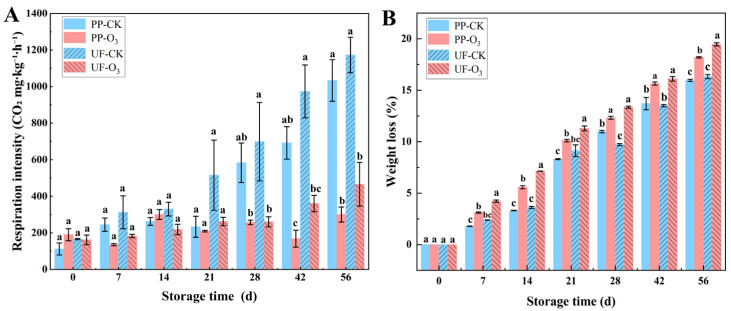
Effects of O_3_ treatment on respiratory intensity (**A**) and weight loss (**B**) of *A. sinensis* at different storage stages. Within each storage day, significant differences between UF-CK (uninoculated control) and UF-O_3_ (uninoculated, ozone-treated) or between PP-CK (inoculated control) and PP-O_3_ (inoculated, ozone-treated) are indicated by different lowercase letters (*p* < 0.05).

O_3_ treatment significantly affected the physiological metabolism of *A. sinensis*, as evidenced by a marked reduction in respiratory intensity (Figure 2A). This effect was observed in roots both inoculated with *P. polonicum* and those with natural contamination. For instance, the respiratory intensity of *A. sinensis* was reduced by 70.94% in the O_3_-treated group (PP-O_3_) in comparison with the control group (PP-CK). In the absence of inoculation with *P. polonicum*, the respiration intensity of *A. sinensis* was also found to be diminished by 60.31% in the O_3_-treated group (UF-O_3_) in comparison with the control group (UF-CK).

During the whole storage period, the weight of *A. sinensis* presented a downward trend, especially with the O_3_-treated group, showing a greater decrease in weight compared to the control group (Figure 2B). This may be explained by the high disease incidence of *A. sinensis* in the control group and the increased humidity. The O_3_ treatment was found to inhibit the growth of a fungus that causes rot in *A. sinensis*, thereby explaining the observed weight loss in the O_3_-treated group, which was found to be higher than in the control group.

**Figure 3:**
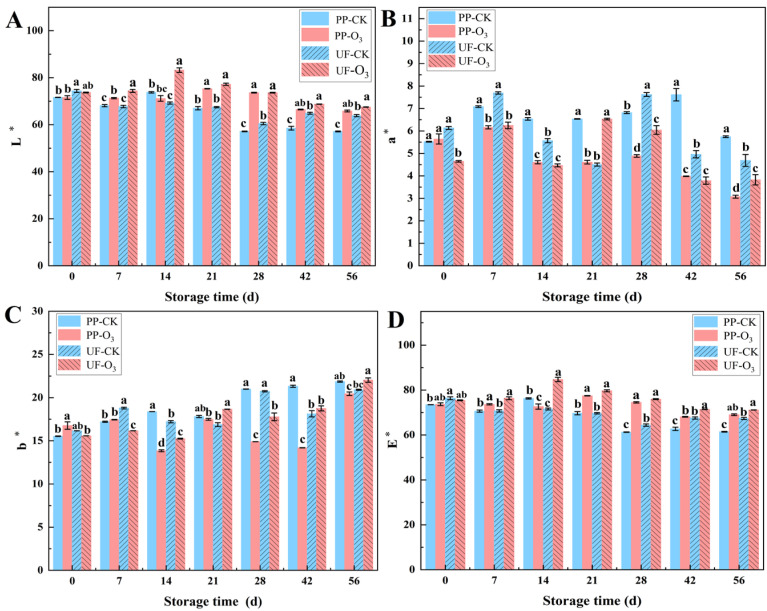
Effects of O_3_ treatment on chrominance of *A. sinensis* during storage. Within each storage day, significant differences between UF-CK (uninoculated control) and UF-O_3_ (uninoculated, ozone-treated) or between PP-CK (inoculated control) and PP-O_3_ (inoculated, ozone-treated) are indicated by different lowercase letters (*p* < 0.05). Subfigures represent different color parameters: (**A**) L* value, (**B**) a* value, (**C**) b* value, (**D**) E* value.

The L* value of *A. sinensis* in the O_3_-treated group exhibited a marked increase in comparison to the control group (Figure 3A), suggesting that O_3_ treatment effectively hindered the darkening of *A. sinensis*. However, O_3_ treatment resulted in a lower a* value when compared to the control group (Figure 3B), and significant variations in the b* values were observed (Figure 3C). The total chrominance of *A. sinensis* demonstrated an initial tendency to increase, followed by a subsequent decrease throughout the storage period. It is noteworthy that the total chrominance of *A. sinensis* in the O_3_-treated group exhibited a greater similarity to the level observed at the commencement of storage (Figure 3D). This finding suggests that O_3_ treatment is efficacious in maintaining the total chrominance of *A. sinensis* during the postharvest storage stage.

#### 3.1.2. O_3_ Treatment Decreased Disease Development

**Figure 4:**
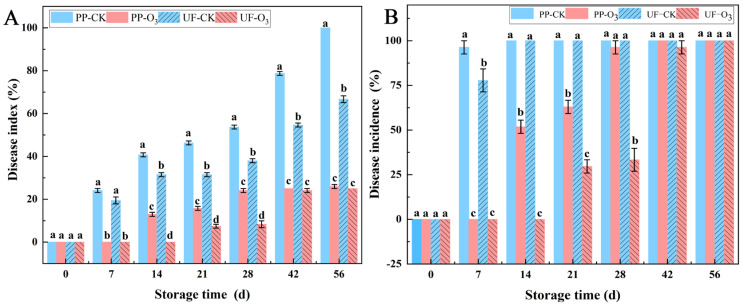
Effects of O_3_ treatment on disease index (**A**) and disease incidence (**B**) of blue mold on *A. sinensis* during storage. Within each storage day, significant differences between UF-CK (uninoculated control) and UF-O_3_ (uninoculated, ozone-treated) or between PP-CK (inoculated control) and PP-O_3_ (inoculated, ozone-treated) are indicated by different lowercase letters (*p* < 0.05).

The application of O_3_ markedly suppressed the development of *A. sinensis* blue mold, thereby extending its storage duration (Figure 4). For instance, the disease index (A) and disease incidence (B) in the PP-O_3_ and UF-O_3_ groups were found to be significantly lower than those in the PP-CK and UF-CK groups. In the control group, the disease index of *A. sinensis* blue mold began on the 7th day of storage and then increased significantly with the extension of the storage period, reaching a peak at the 56th day (PP-CK: 100%; UF-CK: 66.67%). The disease incidence reached its highest point at the 14th day of storage (PP-CK: 100%; UF-CK: 100%). In the O_3_-treated group, the disease symptoms of *A. sinensis* manifested gradually by the 14th day of storage, and the development of lesions was slower. The disease index of the PP-O_3_ and UF-O_3_ groups reached a peak at the 56th day of storage, which was 25.93% in the PP-O_3_ group, a decrease of 74.07% compared with the PP-CK group, and 25% in the UF-O_3_ group, a decrease of 41.67% compared with the UF-CK group.

#### 3.1.3. O_3_ Treatment Maintained the Contents of the Functional Active Ingredients

**Figure 5:**
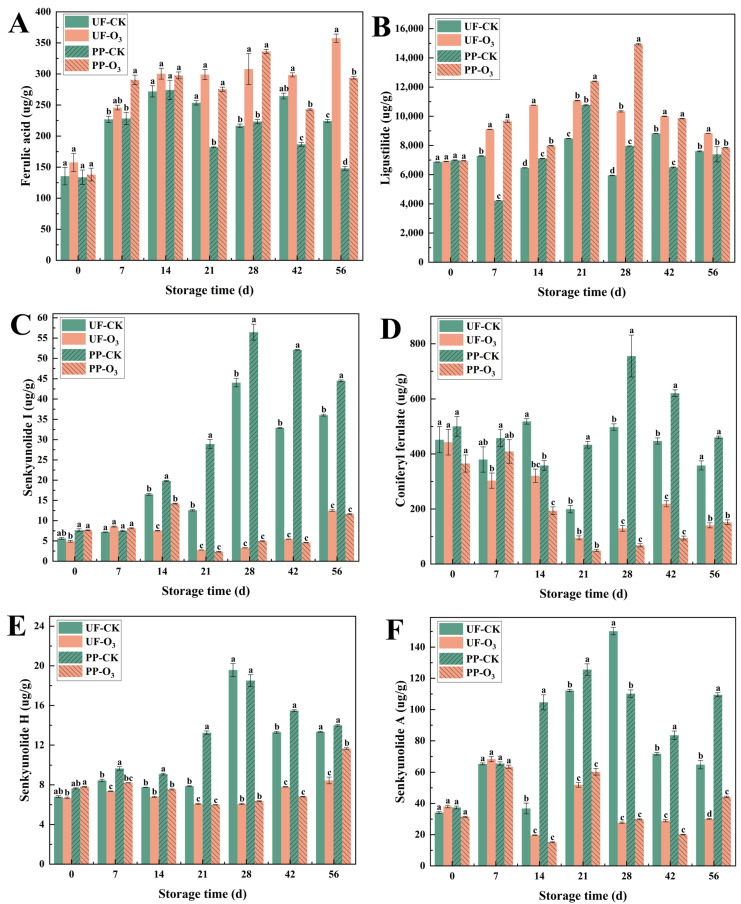
Contents of 6 functional active ingredients of *A. sinensis*. Within each storage day, significant differences between UF-CK (uninoculated control) and UF-O_3_ (uninoculated, ozone-treated) or between PP-CK (inoculated control) and PP-O_3_ (inoculated, ozone-treated) are indicated by different lowercase letters (*p* < 0.05) (ferulic acid (**A**); ligustilide (**B**); senkyunolide I (**C**); coniferyl ferulate (**D**); senkyunolide H (**E**); senkyunolide A (**F**)).

The contents of six major functional active ingredients (ferulic acid, ligustilide, coniferyl ferulate, senkyunolide A, senkyunolide H and senkyunolide I) in *A. sinensis* tissues were determined by UHPLC (Figure S1). The results revealed that the O_3_-treated group exhibited higher levels of ferulic acid and ligustilide (ferulic acid and ligustilide are specified as index components for quality control of *A. sinensis* in the 2020 edition of the Chinese Pharmacopoeia), while coniferyl ferulate, senkyunolide A, senkyunolide H and senkyunolide I levels were reduced in the O_3_-treated group compared to the control group (Figure 5).

### 3.2. Effect of O_3_ Treatment on Metabolites and Metabolic Pathways Related to Disease Resistance of A. sinensis

#### 3.2.1. PCA, PLS-DA and OPLS-DA Analyses

**Figure 6:**
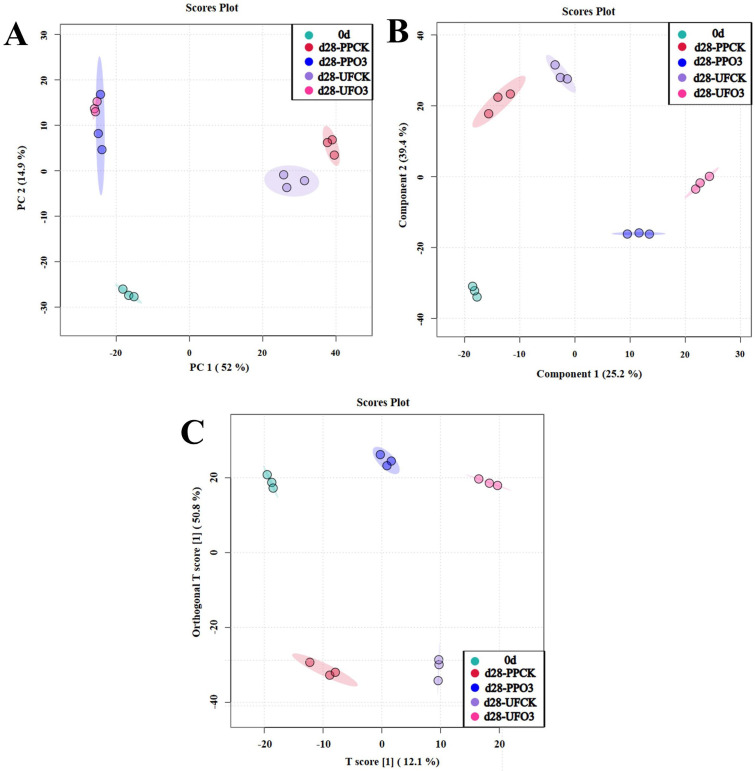
PCA plots (**A**); PLS-DA point cloud (**B**); OPLS-DA point cloud (**C**) of different treatments of *A. sinensis* (PP—inoculated with *P. polonicum*; UF—uninoculated with *P. polonicum*; O_3_—ozone treatment; CK—without ozone treatment; d28 indicates 28 days of storage).

Principal component analysis (PCA) was applied to assess the overall metabolic variation among groups. A score plot (Figure 6A) showed a separation trend along the first principal component (52% of variance) between the ozone-treated (PP-O_3_ and UF-O_3_) and control groups (PP-CK and UF-CK), while overlap within these pairs indicated similar responses to the same treatment. As PCA is exploratory, supervised orthogonal partial least squares discriminant analysis (OPLS-DA) was used to statistically validate the group separation. The OPLS-DA model (Figure 6C) exhibited clear clustering, high explanatory power (R2Y = 0.992), and strong predictive ability (Q2 = 0.907). Its robustness was confirmed by a significant permutation test result (*n* = 200, *p* < 0.01), providing solid statistical evidence that the metabolic profiles of ozone-treated and control groups are distinct. The PLS-DA model showed a consistent pattern (Figure 6B).

#### 3.2.2. Statistics of Metabolite Content

**Figure 7:**
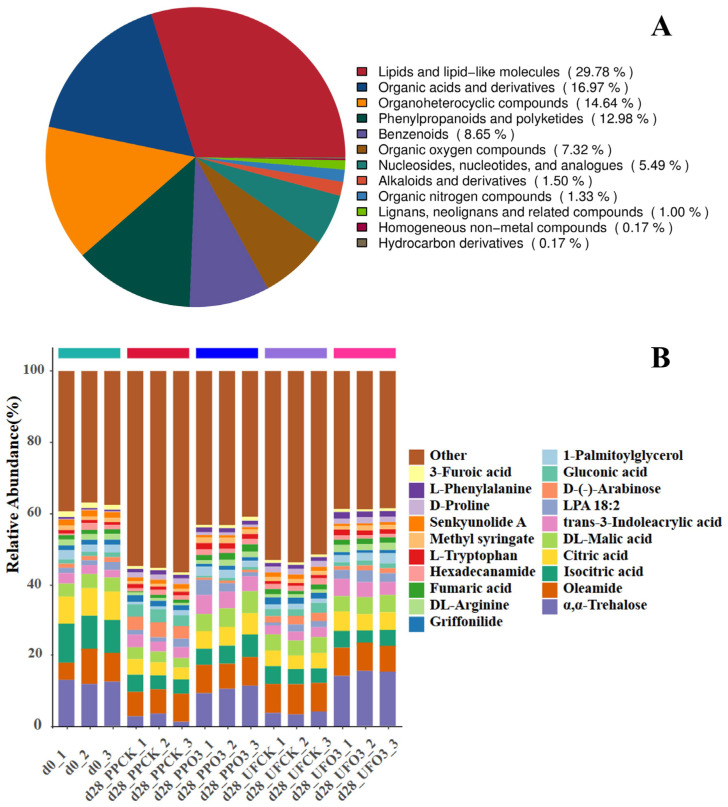
Pie chart of metabolite percentage content (**A**); stacked bar chart of metabolite percentage content of top 20 (**B**) (PP—inoculated with *P. polonicum*; UF—uninoculated with *P. polonicum*; O_3_—ozone treatment; CK—without ozone treatment; d28 indicates 28 days of storage).

A total of 1570 features were detected and putatively annotated (Figure 7A). Analysis revealed that lipids and lipid-like molecules were the most abundant class (29.78%), followed by organic acids and derivatives (16.97%), organoheterocyclic compounds (14.64%), and phenylpropanoids and polyketides (12.98%). The remaining compounds comprised benzenoids (8.65%); organic oxygen compounds (7.32%); nucleosides, nucleotides and analogues (5.49%); alkaloids and derivatives (1.50%); organic nitrogen compounds (1.33%); and lignans and related compounds (1.00%), with homogeneous non-metal and hydrocarbon derivatives each at 0.17%.

The stacked bar chart of the percentage of metabolites with the top 20 contents (Figure 7B) enables a visual comparison of the structural differences in metabolite composition between the comparison groups. The results showed that the top 20 metabolites of *A. sinensis* included L-phenylalanine, D-proline, senkyunolide A, citric acid, isocitric acid, and fumaric acid. We also found that the metabolite contents in the O_3_-treated group were closer to the metabolite contents on day 0, which indicated that the O_3_ treatment was able to better maintain the metabolites of *A. sinensis* tissues.

#### 3.2.3. Metabolite Comparison Between Groups

In this study, a *t*-test *p* < 0.05 and metabolites with VIP > 1 in OPLS-DA indicated differential metabolites between groups. The magnitude of change in differential metabolites was further measured by calculating the fold change (FC) of the metabolites, which was combined with the *p*-value to screen for some metabolites of interest. Using the control group as a reference, the fold change between the mean of the treatment group and the mean of the control group was calculated, with an up-regulation of the fold change being positive and a down-regulation of the fold change being negative. Metabolites with *p* < 0.05 and ∣FC∣ ≥ 2 were considered to be significantly accumulated in different comparison groups. These metabolites were found to be highly variable and should be focused on in this research.

Between-group comparisons were performed to reveal metabolite changes and their involvement in metabolism in *A. sinensis* inoculated with *P. polonicum* after O_3_ application. Specifically, the differential metabolites between d28PPO_3_-vs.-d28PPCK reflected the metabolic changes of O_3_ treatment on *P. polonicum* infection of *A. sinensis* (Table S1). d28UFO_3_-vs.-d28UFCK reflected the effect of O_3_ treatment on metabolic changes associated with the control of naturally occurring diseases during postharvest storage of *A. sinensis* (Table S3).

##### Metabolic Changes in the O_3_ Treatment to Control Blue Mold of *A. sinensis* During Postharvest Storage

**Figure 8:**
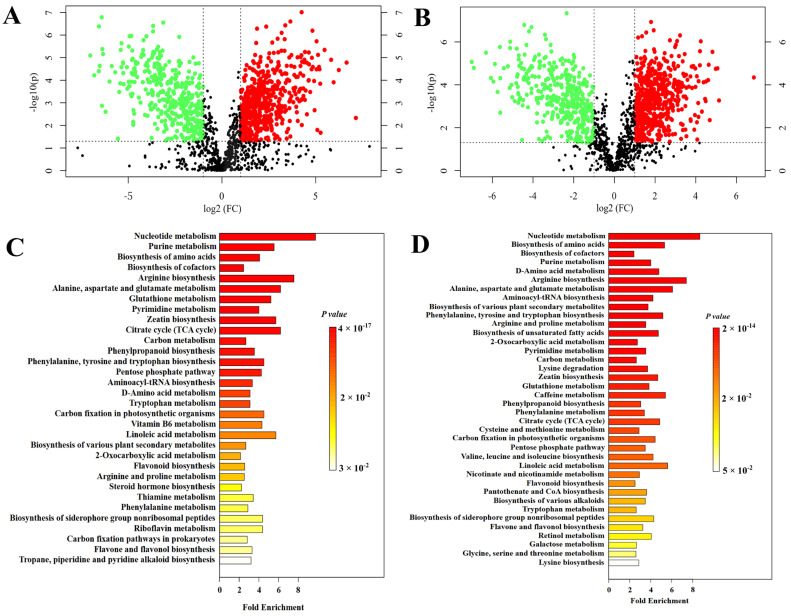
d28PPO_3_-vs.-d28PPCK differential metabolite volcano plot (**A**); differential metabolic pathway enrichment plot (**C**); d28UFO_3_-vs.-d28UFCK differential metabolite volcano plot (**B**); differential metabolic pathway enrichment plot (**D**).

In the d28PPO_3_-vs.-d28PPCK comparison group, 1075 metabolites had significantly accumulated, among which, 625 metabolites were up-regulated, including 552 metabolites with ∣FC∣ ≥ 2; 450 metabolites were down-regulated, including 388 metabolites with ∣FC∣ ≥ 2 (Figure 8A). The fold change in differential metabolites was calculated as the log2 of their differences. It was found that ferulic acid and ligustilide were significantly up-regulated in the d28PPO_3_ group (Table 4), which was consistent with our previous analysis of functional active ingredient compounds of *A. sinensis* using high-performance liquid chromatography (Figure 5).

Differential metabolites with ∣FC∣ ≥ 2 were subjected to KEGG enrichment analysis, and a total of 592 metabolites were assigned to 115 pathways, of which 32 pathways were significantly different (Figure 8C). The number of differential metabolites specifically enriched in each pathway, the up-regulated or down-regulated changes, and the impact values in the topological analysis are shown in Table S2. The differential metabolites enriched in these pathways varied; however, it is noteworthy that the differential metabolites that were enriched in the citrate cycle (TCA cycle) pathway, the phenylpropanoid biosynthesis pathway, the biosynthesis of siderophore group nonribosomal peptides pathway, and the carbon fixation pathways in prokaryotes were all up-regulated, suggesting an up-regulation of these pathways. On the other hand, the differential metabolites that were enriched in the flavone and flavonol biosynthesis pathway and the linoleic acid metabolism pathway were all significantly down-regulated, suggesting a down-regulation of this pathway.

**Figure 9:**
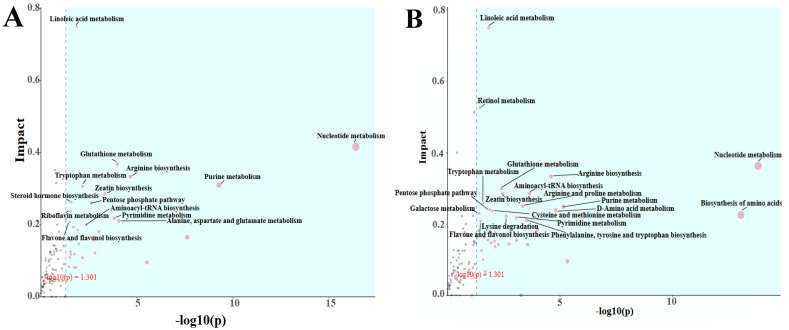
d28PPO_3_-vs.-d28PPCK topology analysis (**A**); d28UFO_3_-vs.-d28UFCK topology analysis (**B**).

The blue area in the topological analysis plot (Figure 9A) indicates the metabolic pathways that were significant in the enrichment analysis. The vertical coordinate, which represents the impact of these metabolic pathways in the topological analysis, is used to measure the criticality of the metabolic pathways that play a role in O_3_-treated *A. sinensis* with blue mold. The results showed 14 metabolic pathways with an impact ≥0.2 out of 32 metabolic pathways with significant differences. These pathways comprised linoleic acid metabolism, nucleotide metabolism, glutathione metabolism, arginine biosynthesis, purine metabolism, tryptophan metabolism, zeatin biosynthesis, steroid hormone biosynthesis, pentose phosphate pathway, riboflavin metabolism, pyrimidine metabolism, alanine, aspartate, glutamate metabolism, flavone and flavonol biosynthesis, and the aminoacyl–tRNA biosynthesis pathway.

##### Metabolic Changes in the O_3_ Treatment to Control of Natural Incidence of *A. sinensis* During Postharvest Storage

There were 1164 metabolites with significant accumulation in the d28UFO_3_-vs.-d28UFCK comparison group; 700 metabolites were up-regulated, of which 586 metabolites were ∣FC∣ ≥ 2, and 464 metabolites were down-regulated, of which 382 metabolites were ∣FC∣ ≥ 2 (Figure 8B). The fold change in differential metabolites was calculated as the log2 of their differences. It was found that ferulic acid and ligustilide were also significantly up-regulated in the d28UFO_3_ group (Table 4), which was completely consistent with the results for the functional active ingredients of *A. sinensis* in our UHPLC analysis (Figure 5).

The differential metabolites with ∣FC∣ ≥ 2 were subjected to KEGG enrichment analysis, and a total of 631 metabolites were enriched into 116 metabolic pathways, of which 38 metabolic pathways were found to be significantly different (Figure 8D). The number of differential metabolites specifically enriched in each pathway, the up-regulated or down-regulated changes, and the impact values in the topological analysis are shown in Table S4. The metabolic pathways in which the enriched differential metabolites were up-regulated comprise the alanine, aspartate and glutamate metabolic pathways; lysine degradation; zeatin biosynthesis; phenylalanine metabolism; the citrate cycle (TCA cycle) pathway; carbon fixation in photosynthetic organisms; the pentose phosphate pathway; nicotinate and nicotinamide metabolism; tryptophan metabolism; the biosynthesis of siderophore group nonribosomal peptides pathway; retinol metabolism; and lysine biosynthesis pathway. Conversely, the differential metabolites involved in the linoleic acid metabolism pathway were found to be down-regulated.

Topological analyses indicated that 19 of the 39 metabolic pathways exhibited significant differences with an impact ≥0.2 (Figure 9B). These metabolic pathways encompass linoleic acid metabolism, retinol metabolism, nucleotide metabolism, arginine biosynthesis, glutathione metabolism, aminoacyl–tRNA biosynthesis, zeatin biosynthesis, tryptophan metabolism, arginine and proline metabolism, purine metabolism, the pentose phosphate pathway, D-amino acid metabolism, cysteine and methionine metabolism, galactose metabolism, biosynthesis of amino acids, lysine degradation, pyrimidine metabolism, phenylalanine, tyrosine and tryptophan biosynthesis, and the flavone and flavonol biosynthesis pathways.

##### Effect of O_3_ Treatment on Disease Resistance in *A. sinensis*

**Figure 10:**
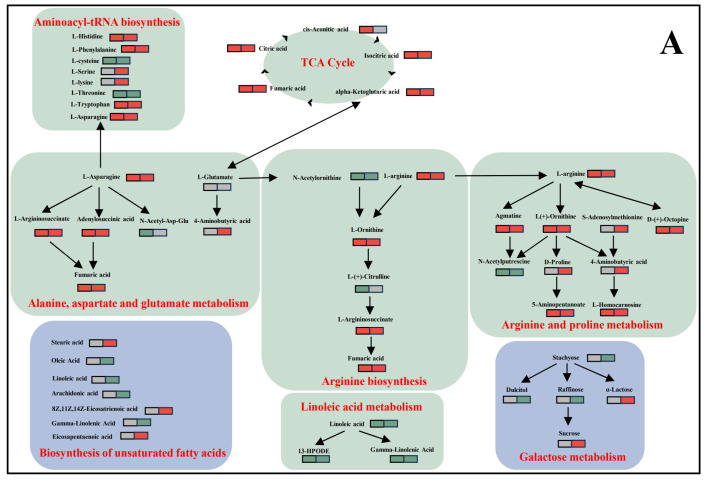

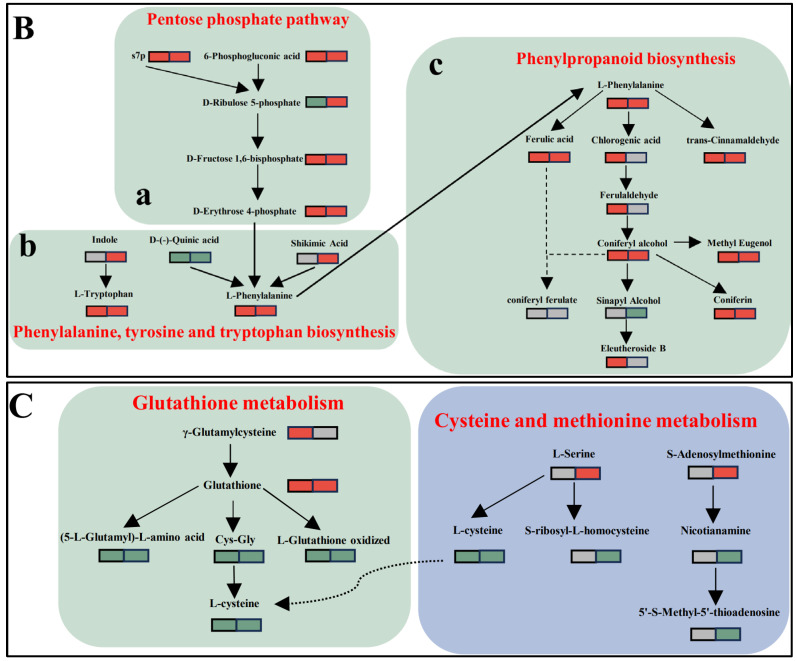
Metabolic pathway map of metabolite changes in the comparison group. (**A**) Schematic diagram focusing on 8 key representative metabolic pathways. (**B**) Schematic diagram focusing on 3 key representative metabolic pathways. (**C**) Schematic diagram focusing on 2 key representative metabolic pathways. (Red indicates that the metabolite was up-regulated to accumulate in the comparison group, green indicates down-regulated accumulation, and gray indicates that this differential metabolite was not enriched in this comparison group. The d28PPO_3_-vs.-d28PPCK comparison group is shown on the left, and the d28UFO_3_-vs.-d28UFCK comparison group is shown on the right. For example, 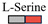 indicates that L-serine was not significantly enriched in the d28PPO_3_-vs-d28PPCK comparison group and up-regulated to accumulate in the d28UFO_3_-vs.-d28UFCK comparison group). (The identification data for the core metabolites shown in the figure are derived from Table S5).

The major metabolic pathways impacted by O_3_ treatment in *A. sinensis* disease resistance are presented in Figure 10. The up-regulation of α-ketoglutarate in the TCA cycle exerts a significant influence on alanine, aspartate and glutamate metabolism, which represents a pivotal step in the linkage of the pathways of each amino acid, which has a further impact on metabolic pathways, including arginine and proline metabolism, aminoacyl-tRNA biosynthesis, arginine biosynthesis, and other metabolic pathways. The linoleic acid pathway was found to be down-regulated, as evidenced by a decline in linoleic acid, 13-HPODE, and γ-linolenic acid content. Furthermore, the biosynthesis of the unsaturated fatty acids pathway and the galactose metabolic pathway was enriched in d28UFO_3_-vs.-d28UFCK (Figure 10A).

Up-regulation of D-erythrose-4-phosphate in the pentose phosphate pathway promotes the biosynthesis of phenylalanine, which in turn is involved in the production of phenylalanine, tyrosine, and tryptophan. Phenylalanine serves as the starting point or switch of the phenylpropanoid biosynthesis pathway, which generates phenylpropanoid metabolites such as ferulaldehyde, coniferyl alcohol, ferulic acid, chlorogenic acid, eleutheroside B, trans-cinnamaldehyde, methyl eugenol, and coniferin through a series of biochemical reactions. These metabolites function as various bioactive effects, such as antimicrobial, antioxidant, and immunomodulatory effects, which improve the resistance of *A. sinensis* against disease. Ferulic acid, chlorogenic acid, ferulaldehyde, and coniferyl alcohol are the main functional active ingredients of *A. sinensis*, and their up-regulation in the O_3_-treated group indicates that O_3_ treatment not only improves the resistance of *A. sinensis* against disease by accumulating antimicrobial metabolites but also maintains the functional active ingredients of *A. sinensis* (Figure 10B).

Reduced glutathione expression was found to be up-regulated in the glutathione metabolic pathway and cysteine and methionine metabolic pathways, and S-adenosylmethionine was up-regulated in d28UFO_3_-vs.-d28UFCK, while L-cysteine was down-regulated (Figure 10C).

In addition to the aforementioned metabolic pathways, topological analyses showed that nucleotide metabolism, tryptophan metabolism, zeatin biosynthesis, pyrimidine metabolism, and the flavone and flavonol biosynthesis pathways were significantly enriched in d28PPO_3_-vs.-d28PPCK and d28UFO_3_-vs.-d28UFCK, which suggests that these pathways are also crucial for the O_3_ treatment to affect *A. sinensis* disease resistance.

## 4. Discussion

### 4.1. Effect of O_3_ Treatment on Physiological, Pathological and Functional Active Ingredients of Fresh A. sinensis

Blue mold caused by *P. polonicum* infection is the most prevalent and important disease in *A. sinensis* during postharvest storage and seriously affects the edible and medicinal value of fresh *A. sinensis* [16]. O_3_ is a food-grade fungicide that protects fruits and vegetables from fungal infections [35,36,37]. In the present study, O_3_ treatment was found to have a significant impact on the infection of *P. polonicum* on *A. sinensis* tissues. The disease incidence and disease index of *A. sinensis* blue mold were reduced (Figure 5), and the occurrence of postharvest diseases in *A. sinensis* was controlled. These results are consistent with those of Zhang et al., who indicated that O_3_ treatment prevented the development of postharvest blue mold in *Lanzhou Lily* [38].

Respiration in postharvest produce is directly linked to energy consumption and nutrient degradation. Higher respiratory rates accelerate quality loss, leading to increased decay and shortened storage life [39]. Conversely, reducing respiration can delay deterioration and help maintain quality [40]. In this study, the respiratory intensity of O_3_-treated *A. sinensis* was significantly lower than that of the control after 14 days of storage (*p* < 0.05; Figure 2A). This result is supported by multiple reports. For instance, Chen et al. found that an appropriate O_3_ concentration (15.008 mg·m^−3^) reduced respiration and decay in *Cantaloupe*, thereby improving its postharvest quality [41]. Similarly, Lin et al. reported that O_3_ treatment effectively suppressed the respiration rate of fresh-cut *water fennel* [42]. Infection by *P. polonicum* typically causes mold development, tissue rot, and darkening in *A. sinensis.* Correspondingly, O_3_ treatment, which reduced disease severity, also resulted in significantly higher L* values (indicating better color preservation) compared to the control (Figure 3A). This further corroborates the efficacy of O_3_ in controlling postharvest blue mold.

In this study, the weight loss in the O_3_-treated group was found to be significantly higher than that in the control group throughout the storage period. This observation may be explained by two factors. First, the increasing disease and gradual increase in rotting of *A. sinensis* in the control group during storage could lead to tissue maceration, resulting in lower measured weight loss than that of the O_3_-treated group. Second, postharvest weight loss in produce is also related to the species of the horticultural products [43]. For example, Bulu et al. found an increase in weight loss in *Pomegranate* during the second month of storage after O_3_ treatment [44]. Similarly, Cayuela et al. demonstrated that O_3_ treatment resulted in a significant increase in the postharvest weight loss of fresh *grapes* after 30 days of refrigeration [45]. These results are in agreement with the findings of the present study.

O_3_ treatment has been shown to play a significant role in the maintenance of functional active ingredients during postharvest storage of *A. sinensis*. Ligustilide, the main active ingredient of *A. sinensis*, helps alleviate oxidative stress and cellular damage induced by external stresses by increasing the expression of various antioxidant genes and inhibiting ROS production via stimulation of the Nrf2/ARE pathway [46]. Senkyunolide A, senkyunolide H and senkyunolide I can be produced via the transformation of ligustilide [47], whose contents were significantly lower, with the content of ligustilide being much greater than that of the transformation products. The reduced levels of senkyunolide A, senkyunolide H and senkyunolide I in the O_3_-treated group may be attributable to the inhibition of the transformation process of ligustilide by O_3_ treatment. Ferulic acid is an important index ingredient for quality control of *A. sinensis* in the 2020 edition of the *Chinese Pharmacopoeia* [14], and it not only has good antioxidant activity [48] but also has a variety of physiological functions, such as anti-inflammatory, antimicrobial, anticancer, antiarrhythmic, and antithrombotic activities. Ferulic acid can be converted into coniferyl alcohol in plants, and coniferyl alcohol is then polymerized by oxidase/laccase to generate lignin, which is the main structural component of the plant cell wall and acts as a physical barrier against pathogenic fungi [49]. It was also reported that there is a process in *A. sinensis* whereby ferulic acid and coniferyl alcohol are converted into coniferyl ferulate and that this process reduces the synthesis of lignin [33]. Therefore, we speculate that the lower level of coniferyl ferulate in this study may be due to the fact that O_3_ treatment inhibited the conversion of ferulic acid and coniferyl alcohol into coniferyl ferulate (Figure 10B(c)), leading to the accumulation of ferulic acid and coniferyl alcohol. This accumulation likely promoted the synthesis of lignin, thereby improving the resistance of *A. sinensis* against disease.

### 4.2. Non-Targeted Metabolomics Analyses of the Mechanism of O_3_ Treatment for the Inhibition of Postharvest Diseases in A. sinensis

The non-targeted metabonomic investigation of O_3_-treated *A. sinensis* resistance to *P. polonicum* infection is essential to discovering its metabolic pathology and exploring new strategies for postharvest disease prevention. The differential metabolites of d28PPO_3_-vs.-d28PPCK and d28UFO_3_-vs.-d28UFCK reflected the effect of O_3_ treatment on the disease resistance of *A. sinensis*. KEGG enrichment analysis of these differential metabolites revealed an up-regulation of organic acids and their derivatives (e.g., cis-aconitic acid, citric acid, α-ketoglutaric acid and fumaric acid) in the TCA cycle of the comparison group (Figure 10A). Among them, citric acid has been demonstrated to effectively regulate the physiological response of plants under different environmental stress conditions [50]. For instance, Ehsan et al. demonstrated that exogenously applied citric acid alleviated the stress of Cd on *Brassica napus* L., which was achieved by reducing oxidative stress (by reducing MDA and H_2_O_2_ production and electrolyte leakage induced by Cd stress) [51]. Similarly, Momma et al. demonstrated that organic acids possess effective antimicrobial properties and can be utilized for the control of plant diseases [52]. The citric acid cycle intermediate α-ketoglutarate is converted into glutamate [53]. Glutamate is a pivotal substance that connects various amino acid pathways and affects the pathways of alanine, aspartic acid and glutamate metabolism; arginine and proline metabolism; aminoacyl-tRNA biosynthesis; arginine biosynthesis; and purine metabolism. A variety of amino acids and their derivatives enriched in these metabolic pathways were up-regulated in the O_3_-treated group, such as L-phenylalanine, L-asparagine, L-histidine, L-arginine, L-tryptophan, L-ornithine, L-serine, L-lysine, and D-proline. The accumulation of these amino acids in plants facilitates the scavenging of excess reactive oxygen species (ROS) and helps maintain redox homeostasis, thus improving disease resistance [54]. In the linoleic acid metabolic pathway, linoleic acid, 13-HPODE, and γ-linolenic acid have all been shown to be down-regulated. Furthermore, research has demonstrated that a variety of fungi have the capacity to produce γ-linolenic acid [55]. Pu et al. demonstrated that linoleic acid is the key metabolite for spore production in *Diaporthe citriby* [56]; Xiao et al. also suggested that *Penicillium Link* fungi can produce linoleic acid, γ-linolenic acid, arachidonic acid, and other polyunsaturated fatty acids [57]. Sucrose and α-galactose in the galactose metabolic pathway have been found to be up-regulated, while raffinose and stachyose are down-regulated in the O_3_-treated group. This phenomenon can be attributed to the consumption of sucrose and α-galactose as a carbon source [16,58], or alternatively, the subjection to fungal stress could have enabled α-galactosidase to catalyze the reaction of galactitol with sucrose, resulting in raffinose, which was enriched as a stress signal [59].

The phenylpropanoid derivatives of ferulaldehyde, coniferyl alcohol, ferulic acid, chlorogenic acid, eleutheroside B, trans-cinnamic aldehyde, methyl eugenol, and coniferin in the phenylpropane biosynthesis pathway were significantly up-regulated after O_3_ treatment (Figure 10B). It is well established that the phenylpropanoid biosynthetic pathway plays a pivotal role in the accumulation of lignin and suberin, thereby thickening the cell wall of the host plant and enhancing its resistance against pathogenic fungi [60,61]. Coniferyl alcohol, as a lignin monomer, is biosynthesized and accumulated in the cell wall, prompting the plant to respond rapidly to both biotic and abiotic stress [62]. Phenolic acids and their derivatives, including ferulaldehyde, chlorogenic acid, coniferin, and ferulic acid, have been shown to possess antioxidant activity and to be the primary functional active ingredients of *A. sinensis*, which are closely related to the pharmacological effects of *A. sinensis* [63]. The polyphenolic substance eleutheroside B has been shown to possess significant antioxidant activity and pharmacological effects, including anti-inflammatory and antidepressant properties [64]. Trans-cinnamaldehyde constitutes the primary antifungal constituent of the essential oil of cinnamon [65], which has been shown to possess potent antifungal properties against a range of pathogenic fungi.

As illustrated in Figure 10C, there was an up-regulation of reduced glutathione expression in the glutathione metabolic pathway, an up-regulation of S-adenosylmethionine, and a down-regulation of L-cysteine in the cysteine and methionine metabolic pathways. Reduced glutathione has been shown to reduce reactive oxygen radicals, thereby generating oxidized glutathione for antioxidant function, and is employed as a protective agent against reactive oxygen species [66]. Wu et al. reported that the exogenous application of GSH was demonstrated to alleviate physiological disorders caused by external stress and improve plant resistance [67]. De Gara et al. observed a decrease in glutathione content in tomato leaves infected with the necrotrophic *Botrytis cinerea* and hypothesized that GSH plays an important role in resistance against *B. cinerea* [68]. L-cysteine, a prevalent amino acid, has been hypothesized by Xu et al. to possess the potential to augment the vitality of a specific synthetic reaction in bacteria, thereby enhancing microorganism survivability [69]. Numerous studies indicate that S-adenosylmethionine has antioxidant and anti-inflammatory properties, and it is also used in the treatment of a variety of disorders, such as depression, osteoarthritis, liver injury, and sleep disorders [70].

In addition, topological analyses showed that nucleotide metabolism, tryptophan metabolism, zeatin biosynthesis, pyrimidine metabolism, and flavone and flavonol biosynthesis are also crucial for the O_3_ treatment to induce resistance against disease in *A. sinensis*. In the present study, the results suggested that four flavonoid differential metabolites of quercetin, luteolin, syringetin, and quercetin-3-O-sophoroside were significantly down-regulated and enriched in the pathways of flavone and flavonol biosynthesis in *A. sinensis* blue mold caused by *P. polonicum* after O_3_ treatment, suggesting that flavone and flavonol biosynthesis was blocked, which may be a result of the O_3_ treatment alleviating the fungal stress of *A. sinensis*. It has been shown that some flavonoid metabolites, such as catechins, quercetin, and rutin, are synthesized when plants are subjected to biotic stress, thereby enhancing the physical barrier against fungi invasion [71,72]. The principal differential metabolites enriched in the tryptophan metabolic pathway included L-tryptophan, tryptamine, 3-indoleacetonitrile and indole, which were found to be significantly up-regulated in the O_3_-treated group in comparison with the control group. Tryptophan plays an important role in the regulation of stress tolerance [73], and some studies suggest that exogenous tryptophan treatment can effectively promote the accumulation of growth-regulating substances and biodefense substances in *Oilseed rape*. This is achieved by regulating the expression of genes associated with the synthesis pathway of the growth hormone indole-3-acetic acid and the biosynthesis pathway of indole-derived glucosinolates. Consequently, this enhances the plant’s potential disease resistance [74]. 3-indoleacetonitrile can be used as a precursor substance to synthesize highly antifungal active novel compounds [75]. Tryptamine, a biosynthetic precursor of many natural alkaloids, has a certain inhibitory effect on cancer cells [76]. In the zeatin biosynthesis pathway, trans-zeatin and dihydrozeatin have been found to be up-regulated, with zeatin being a useful tool in improving plant resistance to disease [77]. Lin discovered that zeatin can regulate tomato growth, thereby improving yield [78]. The greater impact of the pyrimidine metabolic pathway on O_3_ treatment in *A. sinensis* may be attributable to the fact that pyrimidine is a constituent of nucleotides, which play important regulatory roles in the growth, development, and heredity of organisms and are metabolic regulators of many nutrients for plant growth [79]. These metabolic pathways are associated with the metabolic response to O_3_ treatment for the control of *A. sinensis* blue mold, and the main metabolites can also be utilized as potential substances of plant immune activators for disease and quality control.

## 5. Conclusions

In this study, the effects of O_3_ treatment on the physiology, pathology and functional active ingredients of *A. sinensis* during postharvest storage were analyzed, and the impact of O_3_ treatment on the metabolic pathways associated with disease resistance of *A. sinensis* was investigated based on non-targeted metabolomics. O_3_ treatment significantly reduced the respiratory intensity, suppressed the color change and maintained the chrominance of fresh *A. sinensis* during the storage period; however, O_3_ application increased weight loss during storage. The disease incidence and disease index of blue mold of *A. sinensis* were reduced, while the ferulic acid and ligustilide contents, two functional active ingredients specified in the pharmacopoeia, were maintained. Collectively, the quality of the fresh *A. sinensis* was enhanced after O_3_ application. O_3_ treatment primarily promoted the accumulation of organic acids and amino acids in *A. sinensis* through up-regulation of the TCA cycle, thus enhancing the antioxidant capacity of *A. sinensis*. In addition, the O_3_ application activated more antimicrobial substance synthesis by promoting the phenylalanine biosynthesis pathway in *A. sinensis* to protect against the invasion of fungi. The contents of ferulic acid and ligustilide were increased in the O_3_-treated group, which was consistent with the results of our UHPLC analysis. However, the specific process of the inter-transformation of functional active ingredients of *A. sinensis* has yet to be fully elucidated. Further exploration and validation of the effects of O_3_ treatment on transformation among the functional active ingredients of *A. sinensis* from chemical and pharmacological perspectives is recommended. This study will assist in the development of postharvest storage and quality control methods for *A. sinensis*.

## Figures and Tables

**Table 1:** Disease classification of postharvest mildew of *A. sinensis*.

Disease Rating	Disease Rating
0	No disease
1	Fibrous root disease area 0~25% or primary root disease area 0~9%
2	Fibrous root disease area 25%~50% or primary root disease area 10~25%
3	Fibrous root disease area is greater than 50% or the primary root disease area 25~50%
4	Primary root disease area greater than 50%

**Table 2:** Regression equation of functional active ingredients of *A. sinensis*.

Reference Substance	Equation of Regression	r^2^
Ferulic acid	y = 26,336x − 8873.4	0.9998
Senkyunolide I	y = 42,982x − 4689.7	0.9998
Senkyunolide H	y = 58,673x – 30,304	0.9998
Coniferyl ferulate	y = 15,978x − 1731.4	0.9997
Senkyunolide A	y = 7597.6x − 2449.6	0.9998
Ligustilide	y = 10,117x − 208,182	0.9993

**Table 3:** Chromatographic gradient elution procedure.

t	A%	B%
0	98	2
1.5	98	2
3	15	85
10	0	100
10.1	98	2
11	98	2
12	98	2

**Table 4:** Quantitative results of ferulic acid and ligustilide in comparison groups (PP—inoculated with *P. polonicum*; UF—uninoculated with *P. polonicum*; O_3_—ozone treatment; CK—without ozone treatment; d28 indicates 28 days of storage).

	Content	Ferulic Acid	log2(FC)	*p*-Value		Content	Ligustilide	log2(FC)	*p*-Value
Group					Group				
d28PPCK		9.9	3.03	<0.001	d28PPCK		87.0	1.32	0.002
d28PPO_3_		80.5			d28PPO_3_		216.7		
d28UFCK		4.8	1.11	<0.001	d28UFCK		71.7	1.19	0.002
d28UFO_3_		10.4			d28UFO_3_		163.0		

## Data Availability

The original contributions presented in the study are included in the article/Supplementary Material, further inquiries can be directed to the corresponding author.

## Supplementary Materials

The following supporting information can be downloaded at https://www.mdpi.com/article/10.3390/foods15030493/s1: Figure S1: UHPLC chromatograms of 6 kinds function active ingredients; Table S1: Differentially Expressed Metabolites Between Group d28PPO_3_-vs.-d28PPCK; Table S2: Differentially Expressed Metabolic Pathways Between Group d28PPO_3_-vs.-d28PPCK; Table S3: Differentially Expressed Metabolites Between Group d28UFO_3_-vs.-d28UFCK; Table S4: Differentially Expressed Metabolic Pathways Between Group d28UFO_3_-vs.-d28UFCK; Table S5: Table of Raw Metabolite Content.
