# Supplementary material for: Non-Targeted Metabolomics Analysis Reveals the Inhibition Mechanism of Ozone Treatment on Postharvest Blue Mold in Angelica sinensis
Source: Foods. 2026 Feb 1;15(3):493. doi: 10.3390/foods15030493 (PMC12897460; doi:10.3390/foods15030493)
Supplement: Supplementary file 1 [file foods-15-00493-s001.zip › Figure S1.pdf]

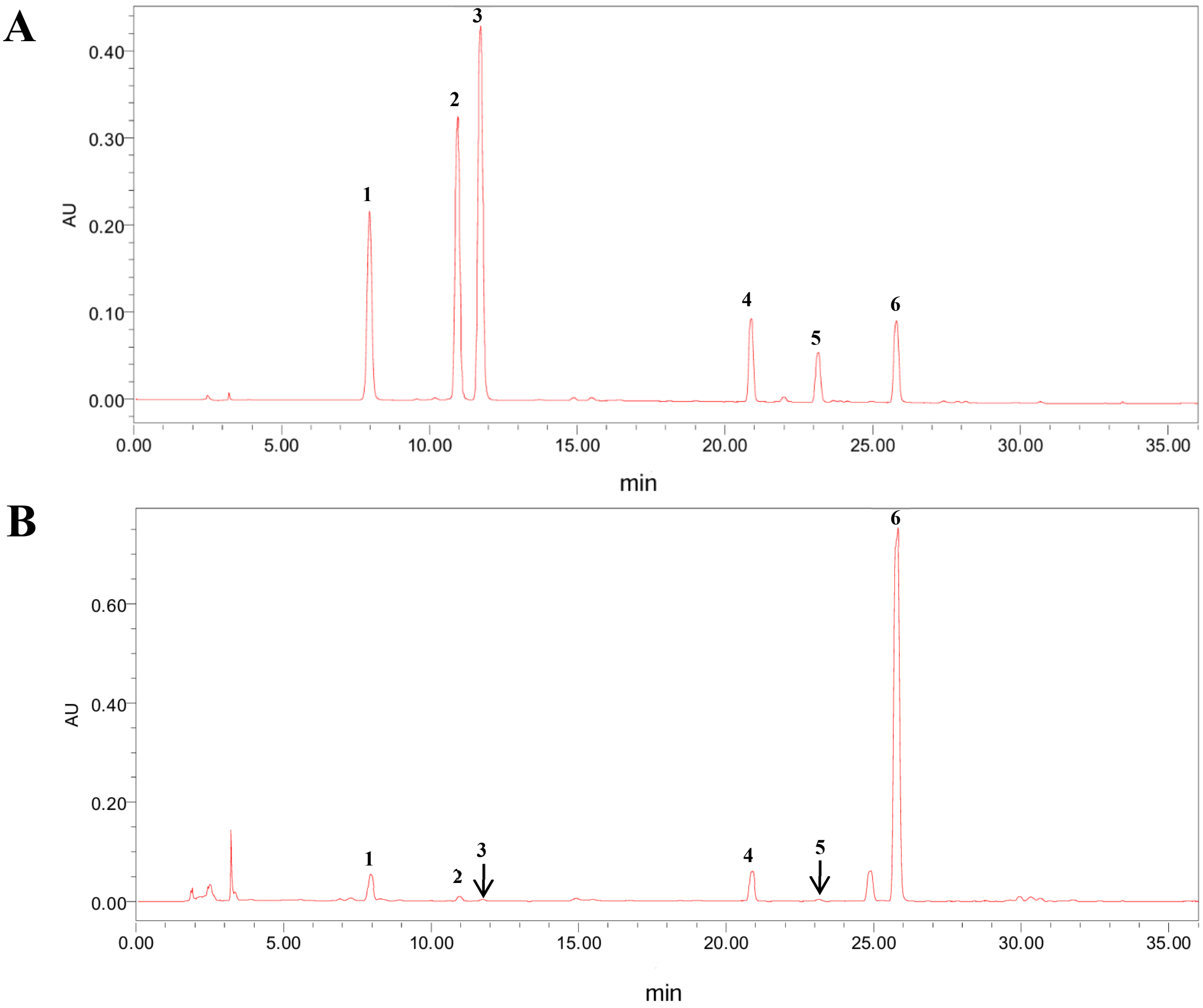

Figure S1. UHPLC chromatograms of 6 kinds function active ingredients (A: standards (100 ug/ml); B: sample (28d-UFCK)

(1-Ferulic acid, 2- Senkyunolide I, 3- Senkyunolide H, 4-Coniferyl ferulate, 5- Senkyunolide A, 6-Ligustilide)
